# An integrative systematic review of creative arts interventions for older informal caregivers of people with neurological conditions

**DOI:** 10.1371/journal.pone.0243461

**Published:** 2020-12-07

**Authors:** J. Yoon Irons, Gulcan Garip, Ainslea J. Cross, David Sheffield, Jamie Bird

**Affiliations:** College of Health, Psychology and Social Care, University of Derby, Derby, United Kingdom; University of Birmingham, UNITED KINGDOM

## Abstract

**Objective:**

We aimed to assess and synthesise the current state of quantitative and qualitative research concerning creative arts interventions for older informal caregivers of people with neurological conditions.

**Methods:**

A systematic search was employed to identify studies that examined creative arts interventions for older informal caregivers, which were synthesised in this integrative review. We searched the following databases: MEDLINE, PubMed, EBSCO, CINAHL, EMBASE, PsycINFO, Cochrane Library, Scopus, Web of Science, and Google Scholar. We also backwards searched references of all relevant studies and inspected trials registers.

**Results:**

Of the 516 studies identified, 17 were included: one was quantitative, nine were qualitative and seven used mixed methods. All included quantitative studies were pilot or feasibility studies employing pre- and post-test design with small sample sizes. Studies varied in relation to the type of creative intervention and evaluation methods, which precluded meta-analysis. Large effect sizes were detected in wellbeing measures following singing and art interventions. The qualitative synthesis highlighted that interventions created space for caregivers to make sense of, accept and adapt to their identity as a caregiver. Personal developments, such as learning new skills, were viewed positively by caregivers as well as welcoming the opportunity to gain cognitive and behavioural skills, and having opportunities to unload emotions in a safe space were important to caregivers. Group creative interventions were particularly helpful in creating social connections with their care-recipients and other caregivers.

**Conclusions:**

The current review revealed all creative interventions focused on caregivers of people living with dementia; subsequently, this identified gaps in the evidence of creative interventions for informal caregivers of other neurological conditions. There are encouraging preliminary data on music and art interventions, however, little data exists on other art forms, e.g., drama, dance. Creative interventions may appeal to many caregivers, offering a range of psycho-social benefits. The findings of the current review open the way for future research to develop appropriate and creative arts programmes and to test their efficacy with robust tools.

## Introduction

The most common neurological disorders are Alzheimer’s disease, Parkinson’s disease, Motor neuron disease, Huntington’s disease and stroke [1]. The causes of these conditions vary, with some having a sudden onset (e.g., spinal cord injury, brain injury), others congenital (e.g., Huntington’s disease), and yet others have unknown causes (e.g., Parkinson’s disease). However, these conditions share common characteristics, such as being long-term and progressively degenerative in nature. Neurological disorders are also the major cause of disabilities worldwide [1] and according to the World Health Organization (WHO), neurological disorders present one of the greatest challenges to public health [2].

People living with a progressive neurological condition require intensive care provision which often means a family member, spouse, or partner becomes their carer. Such informal caregivers or caregivers spend a significant proportion of their day providing unpaid support to the care-recipient. There are 5.5 million caregivers in England and their unpaid work is worth £132 billion, equivalent to the National Health Services (NHS) whole spending, according to the latest report in the UK [3].

Given the chronic nature of a neurological condition, becoming and being an informal caregiver can be a life-changing event, particularly for older adults: the role as a caregiver is new, requiring continuous adjustments [4]. Caregivers may experience days where they can cope, and other days where they feel unable to cope. Caregivers may also feel isolated and lonely [4]. Caregiving is physically demanding and tiring [5]. A recent systematic review found a negative impact of caregiving on caregivers’ health and wellbeing and suggested that effective interventions are needed to reduce these negative impacts of caregiving [6]. Half of caregivers also report they expect worsening quality of life [3]. Thus, the Carers UK report identifies the health and wellbeing of caregivers as an urgent issue for our society [3].

Participating in creative activities (e.g., dance, music, visual arts, etc) have been linked with enhanced wellbeing. Csikszentmihalyi [7] defines creativity as “any act, idea or product that changes an existing domain, or that transforms an existing domain into a new one” (p.28). Being creative is associated with emotional expressiveness [8], and flexible or divergent thinking [9]. Creativity can help caregivers gain different perspectives of the challenges they face and aid them to find or develop new and flexible ways of dealing with those challenges along with helping them to adapt and adjust to the caregiver role [10]. Creative activities offer caregivers opportunities to experience a number of positive psychosocial outcomes, including (i) being connected with others, (ii) being active as they immerse in a creative process, (iii) being reflective of themselves and others through creativity, (iv) learning new skills by exploring and experiencing new things, and (v) experiencing increased self-esteem and empathy [11]. These positive experiences coupled with creative activities are gaining much interest among health professionals and policy makers [12]. Additionally, arts therapists have conceptualised that creativity can build resilience (i.e., the ability to bounce back or recover, through adaptability and flexible thinking). Pivotal agents of resilience, such as empowerment, flexibility, self-efficacy and optimism, can be found, formed and experienced through creative activities [13]. Csikszentmihalyi also makes a connection between creativity and being in ‘flow’, the feeling of being in the moment, experiencing enjoyment and intensive concentration [7]. Such positive experience can further lead to higher life satisfaction, and positively influence the generation of inner resources, such as resilience [14]. For example, Lepp et al. [15] evaluated a drama programme that included dance, rhythm, song, and storytelling for people with dementia and their caregivers at a care home. Through this creative programme, people with dementia and their caregivers shared feelings of joy and sadness, and experienced a sense of belonging and togetherness, which helped communication between them.

At a political level, the potential health and wellbeing benefits from participating in creative programmes have been recognised: the UK All-Party Parliamentary special interest group on Arts, Health and Wellbeing produced a comprehensive review of creative interventions for the health and wellbeing [12]. In this report, the benefits for caregivers are also presented based on both anecdotal and research evidence. Many caregivers take part in participatory arts-based programmes designed for people with a health condition. For example, ‘arts on prescription’ programmes provided creative and participatory workshops, which have the potential to positively influence health and wellbeing [12]. Further, WHO’s recent report on dementia revealed that by 2030 we can expect around 75 million people worldwide would live with dementia, and the report highlighted that more support for their informal/family caregivers is needed [16]. Given the growing evidence of creative arts interventions for informal caregivers, and in the absence of a systematic review of this field, we undertook this review using an integrative approach [17], of both quantitative and qualitative research evidence. Our aim was to assess the evidence, identify gaps in the evidence-base, and guide future research, practice and policy concerning the caregivers of people with neurological conditions.

## Methods

The protocol of this review was registered at PROSPERO, an international prospective register of systematic reviews (Registration ID CRD42019129857). We used pre-determined criteria for considering studies to include in the review, in terms of types of studies, participant and intervention characteristics.

### Eligibility criteria

#### Studies

We included empirical studies, descriptive, correlational, qualitative and quantitative studies (randomised controlled trials and non-randomised pre-/post-test) published in peer-reviewed journals. Editorials, position papers, case studies, commentaries, expert reports, abstracts, conference proceedings, and dissertations were excluded. Studies did not have to include a comparator group, but could include comparator groups that completed exercise, or psychotherapy, or had passive (treatment as usual), or wait-list control groups.

#### Participants

This review included studies of informal caregivers (aged >50 years) who cared for a family member with a neurological condition, e.g., Dementia, Parkinson's Disease, Huntington Disease, Multiple Sclerosis, Motor Neuron Disease and Stroke. Studies involving paid professional caregivers were excluded from this review.

#### Creative arts interventions

Creative arts interventions use mediums such as music, arts, drama, dance, to promote better psychological and physical health and wellbeing. This review focused on creative arts interventions, which contain acts of creation on the part of participants: i.e., based on the definition of creativity by Csikszentmihalyi [7], this review included studies, where participants were actively engaged in making/transforming an art form, or an idea into a new one. (The definition of a creative arts intervention can be found in S1 Appendix.) For example, we included an art-gallery based intervention where people with dementia and their caregivers viewed arts, and then were encouraged to make their own artwork following the viewing sessions [18]. Studies without creative activities were excluded. For example, listening to a playlist of music [19] and viewing arts alone [20] were excluded, as these programmes do not involve making or transforming an art form. An expressive writing study prescribed a strictly defined writing process, where participants were asked to write down only thoughts and feelings about stressful or traumatic life events [21]. This study did not promote creativity: there was no room for divergent thinking nor transformation of feelings through writing. Similarly, those interventions that involved the viewing of painting or photos to tell autobiographical stories were excluded [22].

#### Types of outcome measures

This review assessed the effects of creative arts interventions on:

- Quality of life measures (e.g., SF-36, SF-12; WHOQOL-Bref);
- Wellbeing measures (e.g., ICECAP, WHO-5, or Warwick Edinburgh Mental Wellbeing Scale (WEMWBS));
- Mental health measures (e.g., Geriatric Depression Scale, Patient Health Questionnaire);
- Caregiver burden and stress measures (e.g., Caregiver Burden Scale, Burden Scale for Family caregivers, Perceived Stress Scale)

Similarly, qualitative articles were included if they explored quality of life, well-being or mental health or included discussion of carer burden and stress.

#### Search methods

We determined search terms using the CHIP tool (Context, How, Issues, Population) [23]. We also consulted with a librarian in relation to adapting the search terms for electronic databases searches. We searched the following electronic bibliographic databases: MEDLINE, PubMed, EBSCO, CINAHL, EMBASE, PsycINFO, Cochrane Library, Scopus, Web of Science, and Google Scholar. We also inspected references of all relevant studies; searched trials registers (ClinicalTrials.gov). There were no language or temporal restrictions. We also cross-checked reference lists in the relevant papers and looked for papers that have cited the relevant papers in Google Scholar. The last search was performed on 31^st^ May 2019. For detailed search strategies, please see S2 Appendix.

#### Data extraction and management

Quantitative studies were selected by two (JYI & JB) authors and qualitative studies by another two authors (GG & AC) according to pre-defined inclusion criteria. From eligible papers, data extraction was independently conducted by authors (quantitative–JYI, DS; qualitative–GG, AC), including relevant details, such as study design, objectives, location, length, participants’ demographic data, their family member’s health condition, outcome measures, findings, statistical analysis and qualitative analysis. We extracted quantitative data of pre-/post-tests and follow-up where possible. Any discrepancies were resolved by discussion with a third author (JB).

#### Assessment of study quality

Two teams of authors independently screened included studies for their quality. The Critical Appraisals Skills Programme (CASP) tool [24] was applied to appraise the qualitative data reported (GG & AC), while the Downs and Black tool [25] for quality assessment was used for quantitative data reported (DS & JB). These appraisal tools were selected for use in the present review as they are well-established for use with the respective type of article and the tools have clear guidance for reviewers, as well as ensures transparency and replicability [26, 27].

#### Data analysis methods

For quantitative data, effect sizes were extracted for each study; where effect sizes were reported we used them. If they were not available, then we used means, standard deviations and sample sizes at baseline and post-intervention of experimental (and control, if available) conditions; in one case we extrapolated from data presented in a figure. Where such statistics were missing, we used test statistics including F and *t*–values. If findings were reported as non-significant and no data was reported, we assumed the effect was 0. We planned to conduct a meta-analysis, using random-effects models, if there were more than four studies with comparable outcome measures.

For qualitative studies, we followed the steps for conducting a meta-synthesis in accordance with meta-ethnography from an interpretivist philosophical stance [28]. Whilst we acknowledge there are other approaches to conduct interpretive syntheses (e.g., meta-study, critical interpretive synthesis, etc.) this approach was chosen as it was well-suited to synthesise empirical research conducted within different research paradigms (e.g., medicine, psychology, arts, etc.). The goal of the meta-ethnography was to allow for interpretations that went beyond the individual selected studies. There are 7 phases to conducting a meta-ethnography. Initially the authors established the aim of the review (phase 1), which was to understand the influence of creative interventions for caregivers of people with neurological conditions. In phase 2, after having taken an exhaustive search, we refined the searches to focus on informal caregivers who had taken part in a creative intervention either individually or with the person they were caring for, i.e., the care-recipient. In phase 3, after having selected eligible studies, we read and re-read the studies to develop an understanding of each paper. The next three stages were carried out in parallel, where studies were read to inform lines-of-argument (phase 4) that allowed for presenting a bigger picture of caregivers experiences and involvement in creative interventions informed by each study (phase 5). Data extraction involved the following steps: first (i.e., participant quotes) and second (i.e., paper authors’ interpretations and themes) order constructs from papers included in the review were extracted into matrix where it was possible to categorise themes from papers separately. (An example of this process can be found in S3 Appendix. Audit Trail sheet 1 in S3 Appendix.) Then we examined themes from separate papers to establish related themes from other papers (An example of this process can be found in S3 Appendix. Audit Trail sheet 2 in S3 Appendix.) These categories led to the 6^th^ phase and enabled the development of third order constructs or meta-themes (i.e., review authors’ interpretations were presented in the results section (phase 7)), which synthesised the findings from the eligible papers, in line with the aims of this review.

## Results

### Results of the searches

**Fig 1:**
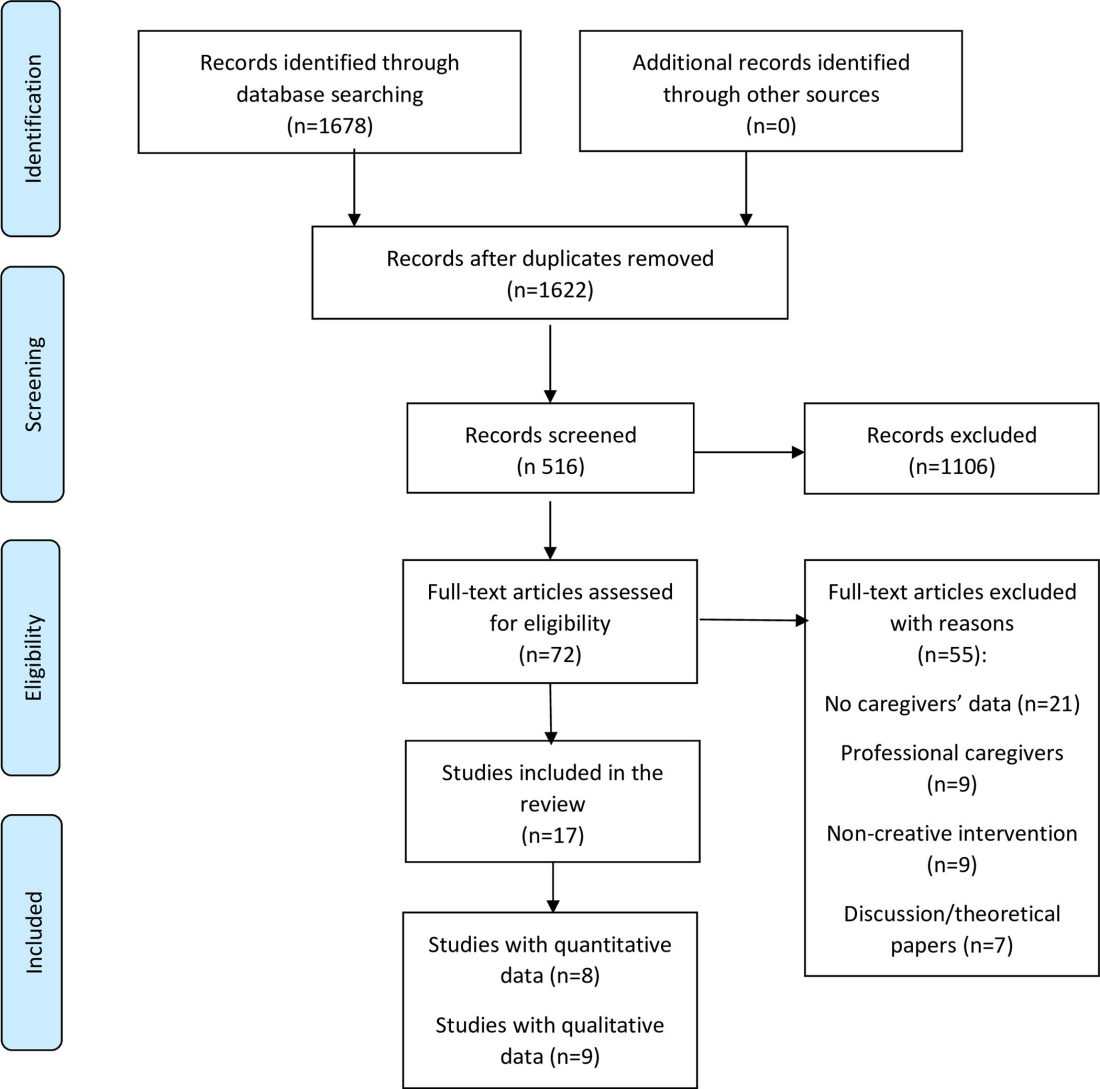
PRISMA flowchart.

After database searches and relevant websites, 1678 hits were recorded. 56 duplicates were removed; 72 full texts were assessed against the inclusion criteria. First, titles and abstracts were screened, and relevant papers were chosen. Subsequently, full texts of those relevant papers were obtained and reviewed carefully. The PRISMA (Preferred Reporting Items System Meta-Analysis) chart is presented in Fig 1.

A total of 17 studies were included: nine studies utilised only qualitative approaches [29–37], one study used only quantitative methods [38] and seven studies employed both qualitative and quantitative measures [18, 39–44]. Included paper characteristics are presented in Table 1. A total of 172 caregivers were included in this review. The creative interventions included singing [29, 30, 34, 39–42, 44], song writing [37, 43], art viewing combined with art making sessions [18, 31, 32, 35], and music combined with art [33, 38]. The length and duration of the creative interventions varied: Bourne and colleagues examined the benefits of each of a single 60-minute singing and art viewing session [38], while Tamplin and colleagues offered a 20-week programme [39]. Others provided weekly programmes for 6 weeks [41, 43, 44], 8 weeks [18] and 10 weeks [42]. The majority of the included studies provided creative interventions to improve health and wellbeing of both people with dementia and their caregivers. Included studies employed relatively small number of caregivers, ranging between three [33] and 21 [36]. All studies recruited caregivers of people with dementia. Seven studies were from UK, six from Australia, three from the USA, and one study from Israel.

**Table 1:** Summary of characteristics of included studies.

Authors Year	Condition of care-recipient	Aims	Intervention	Number of Caregiver participants (age & gender)	Relationship to care-recipient	Data Collection	Data Analysis	Findings
Country								
[Reference number]								
**Baker**, Grocke & Pachana (**2012**)	Dementia	To explore a home-based music programme on the health, wellbeing, and relationship quality of spousal caregivers and their partners with dementia	6- week active music intervention involving training caregivers in music therapy	N = 5; 3 females, (age = 81, 81, 61 years old)	Spouses; Caring for husband = 3; caring for wife = 2	Mixed-methods;	Pre-post mean comparison,	Music sharing experience was beneficial for spousal relationship and satisfaction of caregiving and enhanced mood.
Australia				2 males (age = 74, 59 years old)		Quality of spousal relationship, carers’ wellbeing;	Thematic analysis^a^	
[44]						Diaries kept during the 6-week post-programme; semi-structured interviews		
**Baker** & Yeates (**2017**)	Dementia	Explore carers’ experiences of participating in a creative group song writing process	4-week group song writing facilitated by music therapists and support staff	N = 4; 2 females, 2 males	Caring for mother = 2; caring for wife = 1; caring for husband = 1	Focus group; individual interviews at post- intervention	Interpretative Phenomenological analysis^b^	Co-creating songs were meaningful and empowering for carers.
Australia				Age = NR				
[37]								
**Baker**, Stretton-Smith, Clark, Tamplin & Lee (**2018**)	Dementia	To test the feasibility of implementing a group song writing programme with family caregivers of people living with dementia	6-week, 1-hour song writing sessions facilitated by a trained music therapist with groups of 3–5 participants	N = 14	Family caregivers (11 caring for a spouse; 2 were adult children caring for a parent)	Mixed-methods;	Pre-post mean comparison;	The song writing process was found to be beneficial.
Australia				9 females, 5 males		Patient Health Questionnaire; Quality of the caregiver patient relationship;	Thematic analysis^a^	
[43]				(8 = intervention group; 6 = control group);		Focus groups with caregivers		
				*M*age *=* 72.29 (SD = 6.72 years old)				
**Bourne**, Camic, Crutch, Hulbert, Firth & Harding (**2019**)	Dementia	To assess the impacts of choral singing and art viewing on wellbeing of caregivers	Single 60-minute session of choral singing; single 60-minute session of art viewing	N = 7; 6 females, 1 male	Caring for partners or family members	Heartrate variability; saliva assays (stress hormone); Canterbury Wellbeing Scale; Visual Analogue Scale	Wilcoxon signed-rank tests	No statistical significance between interventions. No statistical significance improvement in measures.
U.K.				*M*age = 60.86 (SD = 11.10 years old)				
[38]								
**Burnside**, Knecht, Hopley & Longsdon (**2015**)	Dementia	To improve quality of life for caregiver and person with dementia through a community-based arts programme	Gallery tours and art classes for dyads	N = 21	Caring for partners	Semi-structured in-depth telephone interviews at 2-week post participation	Grounded theory analysis^c^	High levels of engagement, mindfulness, social connection, and positive interactions.
USA				age = NR				
[36]				gender = NR				
**Camic,** Williams & Meeten (**2011**)	Dementia	To measure the impact of signing on wellbeing and quality of life for people with dementia and caregivers	10-week, 90- minute ‘Singing together group’ facilitated by a community musician	N = 10	9 spousal, 1 daughter caregiver	Quality of life questionnaire; Mental health; Semi-structured interviews with caregivers	Repeated measures *t*-tests	Depression, anxiety and stress remained stable pre- and post-intervention for careers.
U.K.				age = NR; gender = NR			Thematic analysis^a^	Pre-intervention deliberation, ambience and environment, structure, social inclusion, the experience of signing.
[42]								
**Camic**, Tischler & Pearman (**2014**)	Dementia	To understand how programs at contemporary and traditional galleries play a role in the lives of people with dementia.	8-week, 2- hour group sessions, where first hour is art viewing, followed by an hour of art making, supported by gallery facilitators.	N = 12	Caring for partners	Quality of life questionnaire, carers burden interview, activities of daily living scale	Paired *t*-test, Wilcoxon signed-rank tests	No statistical significance between galleries. No statistical significance improvement in measures. 3 themes = social impact; cognitive capacities; Art gallery setting
U.K.				age = NR; gender = NR		Semi-structured Interviews	Thematic analysis^d^	
[18]								
**Camic**, Baker & Tischler (**2015**)	Dementia	To understand how programmes at contemporary and traditional galleries play a role in the lives of people with dementia and their carers	8-week, 2- hour group sessions, where first hour is art viewing, followed by an hour of art making, supported by gallery facilitators	N = 12	Caring for partners	Post-intervention interviews; researchers’ field notes	Grounded theory analysis^c^	The art gallery was a valued place that provides intellectual stimulation and opportunities for social inclusion, and positive emotional and relational effects for the dyad.
U.K.				age = NR; gender = NR				
[35]								
**Clark**, Tamplin & Baker (**2018**)	Dementia	Explored caregivers' perspectives and experiences of group singing intervention	Therapeutic group singing intervention over 20-week	N = 9; 5 females, 4 males	Family caregivers; husbands and wives	Semi-structured dyad interviews	Thematic analysis^a^	Enhanced relationships and social, cognitive and wellbeing for family caregivers
Australia				*M*age = 75.7				
[34]								
**Dassa** (**2018**)	Dementia	To help alleviate caregiver burden during visiting hours.	Creation of an individualised database using personal music and photos that present life episodes for care-recipients	N = 3 females, 59, 75, & 76 years old	Caring for a husband	Pre-intervention interview; Post-intervention interview; Follow-up phone call	Systematic content analysis^e^	Using individualised database helped caregivers find ways to communicate with their partners, relive shared past experiences, and alleviate the stress and feelings of disconnection during visits.
Israel								
[33]								
**Davidson** & Almeida (**2014**)	Dementia	To explore whether engagement in a singing programme could result in improvement stress and mood, for carer and care-recipients	Structured singing session over 6 weeks facilitated by music educators	N = 6	Caring for partner or parent	Mixed methods	Paired Samples *t*-tests	Positive experiences and powerful impact of intervention participation on dyads.
Australia			Group A = community Centre;	*M*age = 69.67 (SD = 16.21 years old); gender = NR		Post-intervention interviews	Not specified for the interview data	
[41]			Group B = Residential facility;					
			Control group = quiz session					
**Flatt**, Liptak, Oakley, Gogan, Varner & Lingler (**2015**)	Alzheimer’s disease or related cognitive disorders	To describe the subjective experience of dyads participating in an art museum activity	One-time 3-hour activity, including 1- hour guided tour and 2- hour art making activity at the art museum	N = 10	Caring for family member	4 focus groups; researchers’ field notes	Thematic analysis^g^	Positive impact on cognitive stimulation, social connections, and self-esteem were identified as key themes.
USA				4 males, 6 females				
[32]				> 60 years old				
**Hanser**, Butterfield-Whitcomb,Kawata & Collins (**2011**)	Alzheimer’s Dementia	To explore the benefits of a caregiver-administered music programme	A music intervention led by caregivers after receiving 2-hour training from a music therapist; carers provided music programmes	N = 14 dyads enrolled, N = 8 dyads completed; 3 males, 5 females 65> n = 2; 65–75 n = 2, 76–85 n = 2, >85 n = 2	Spouse (n = 6); daughters (n = 2)	Mixed methods; carer burden, satisfaction scales	Wilcoxon Signed Ranks test	Increase in reminiscence; increase in household music making and joining in from other family members, enjoyment of music, and criticism of protocol
USA						Semi-structured interviews	Thematic analysis^g^	
[40]								
**Hunt**, Truran & Reynolds (**2018**)	Dementia	Explores carers experiences of leisure-based art making and its contribution to psychological well-being	Women taking part in art interventions in local art, day centres, and community centres.	N = 6	Family caregivers	Semi-structured in-depth interviews	Interpretative phenome-nological analysis^b^	Meaningful leisure occupations may help to protect the psychological wellbeing of caregivers and promoting resilience
U.K.				females, Age range = 60–77 years old				
[31]								
**Osman**, Tischler & Schneider (**2016**)	Dementia	To explore the impact of *Singing for the Brain*^*TM*^ for people with dementia and their carers	A specially developed group singing programme led by a trained musician	N = 10	3 daughters caring for their mother, 5 wives caring for husband, and 2 husbands caring for wives	Semi-structured interviews	Thematic analysis^a^	Social inclusiveness improvement in relationships, memory, and mood
U.K.				8 females, 2 males				
[30]				age = NR				
**Tamplin,** Clark, Lee & Baker (**2018**)	Dementia	To assess the benefits of ‘Remini-sing’ for people with dementia and their family care givers	Weekly 2- hour group singing programme for 20-week; person with dementia and carer dyads; in community setting, led by music therapists	N = 12 dyads enrolled	Family care givers	Mixed methods	Paired sample *t*-tests	No statistically significant changes in measures of carers wellbeing
Australia				*M*age = 73.9 (SD = 10.1 years old)		Carers’ life satisfaction, relationship, mental health; semi-structured interviews at post-intervention	Thematic analysis^a^	
[39]				6 males, 6 females				Singing programmes was well received; difficulties with self-report measures for people with dementia
				9 dyads completed the study				
**Unadkat**, Camic & Vella-Burrows (**2017**)	Dementia	To better understand how group singing benefits people with dementia and their partners.	Various singing groups in England and Wales	N = 17	Caring for a partner	Interviews with couples	Grounded theory approach^f^	Enjoyment of singing experience, stimulating, uplifting, therapeutic and positive effects of the group, couple benefits, and caregiver benefits.
U.K.				1 male, 9 females *M*age = 70.3 (range = 61–89 years old)				
[29]								

NR = Not Reported in the paper; *M*age = Mean age; SD = Standard Deviation

Thematic analysis^a^ = based on Braun & Clarke [45]

Interpretative phenomenological analysis^b^ = based on Smith et al. Interpretative Phenomenological Analysis [46]

Grounded theory analysis^c^ = based on Corbin & Strauss [47]

Thematic analysis^d^ = based on Boyatzis [48]

Systematic content analysis^e^ = based on Kohlbacher [49]

Grounded theory approach^f^ = based on Glaser & Strauss [50]

Thematic analysis^g^ = Authors reported without a reference.

### Quantitative studies

Eight of the included 17 studies employed quantitative measures [18, 38–44], which included a total of 74 caregivers. Creative interventions included in these studies were singing [38, 39, 41, 42], song writing [43], music [40, 44] and art viewing [18, 38]. All eight studies presented findings of an exploratory nature: they included small number of participants (between 5 and 14); the majority studies were experimental with a pre- and post-test design, except two studies [38, 43]: a song writing programme was compared with standard care [43], and group singing with art viewing [38]. Studies examined the impact of a creative programme on improved wellbeing [38–41], mental health (depression, anxiety) [39, 42–44], stress [38, 42], quality of relationship in couples [39, 43], caregivers’ burden [18, 40], satisfaction with caring role/life [39, 44], and quality of life in caregivers [18] (Table 1).

For outcomes of interest, each of the studies’ pre-post effect sizes revealed some benefits of the intervention: Large effect sizes were detected for singing on wellbeing (*d* = 1.04) [38], and positive-negative mood (*d* = 1.29) [41]; and for music making on relaxation (*d* = 1.91), comfort (*d* = 1.74), and happiness (*d* = 1.19) [40] (Table 2). Some studies indicated decreases in some aspects of wellbeing which were not consistent with other aspects reported. For example, Camic [42] reported increases in stress, but decreases in anxiety and depression. (The data used to calculate effect sizes along with where it is reported in the article is uploaded as S4 Appendix.)

**Table 2:** Quantitative studies: Outcome measures and effect sizes.

1^st^ Author, year [Reference number]	Outcome measures	Number of Participants included in the analysis	Effect size d* (95% Confidence Intervals)
**Baker, 2012** [44]	Geriatric Depression Scale Short Form (GDS)	Music n = 5	*d* = 0.35 (-0.92–1.58)
	Geriatric Anxiety Inventory (GAI)		*d* = 0.45 (-0.85–1.65)
	Mutual Communal Behaviours Scale (MCBS)		*d* = 0.46 (-0.82–1.69)
	Positive Aspects of Caregiving Questionnaire (PACQ)		*d* = 0.25 (-1.02–1.47)
	Neuropsychiatric Inventory Questionnaire (NPI)		*d* = 0.41 (-0.84–1.66)
**Baker, 2018** [43]	Measures of Depression (PHQ-9)	Song writing group: n = 8	Song writing: *d* = 0.64 (-0.50–1.49)
		Standard care: n = 6	Control: *d* = -0.33 (-1.56–0.73)
	Perceptions of Caregiving Experience (PACQ)	Song writing: n = 8	Song writing: *d* = -0.24 (-1.30–0.67)
		Standard care: n = 6	Control: *d* = -0.18 (-1.30–0.97)
	Perceptions of Relationship with the Care Recipient (QCPR)	Song writing: n = 8	Song writing: *d* = 0.14 (-0.85–1.11)
		Standard care: n = 6	Control: *d* = 0.57 (-0.47–1.86)
**Bourne, 2019** [38]	Stress (Visual Analogue Scale)	Singing: n = 7	Singing: *d* = 0.59 (-0.55–1.58)
		Art viewing: n = 6	Art viewing: *d* = 0.26 (-0.88–1.39)
	Canterbury Wellbeing Scale	Singing: n = 7	Singing: *d* = 1.16 (-0.20–2.00)
		Art viewing: n = 6	Art viewing: *d* = 0.60 (-0.64–1.66)
**Camic, 2011** [42]	Depression Anxiety Stress Scale (DASS)–Depression	Singing n = 8	*d* = 0.24 (-0.77–1.12)
	–Anxiety		*d* = 0.49 (-0.56–1.42)
	–Stress		*d* = -0.24 (-1.25–0.72)
	WHO Quality of Life Questionnaire (WHO QOL_Bref)	Singing n = 9	
	–Physical		*d* = 0.05 (-0.89–0.96)
	–Psychological		*d* = 0.13 (-0.82–1.03)
	–Social		*d* = 0.28 (-0.70–1.16)
	–Environment		*d* = 0.20 (-0.75–1.10)
**Camic, 2014** [18]	Zarit Burden Interview (ZBI)	Location 1: n = 8	*d* = 0.23 (unknown^)
		Location 2: n = 6	*d* = 0.62 (unknown^)
**Davidson, 2014** [41]	Numeric Rating Scale–Positive-negative mood	Singing (Group A): n = 6	*d* = 1.29 (unknown^)
	–Energised-tired		*d* = 0 (unknown^)
	–Relaxed-stressed		*d* = 0 (unknown^)
	–Focused-unfocused		*d* = 0 (unknown^)
**Hanser, 2011** [40]	Visual Analogue Scale–Relaxation	Music n = 8	*d* = 1.91 (0.86–3.28)
	–Comfort		*d* = 1.74 (0.67–3.01)
	–Happiness		*d* = 1.19 (0.27–2.45)
	Caregiving Satisfaction Scale	Music n = 8	*d* = 0.10 (-0.32. - 0.52)
**Tamplin, 2018** [39]	Quality of Caregiver Patient Relationship (QCPR)	n = 9	*d* = -0.12 (-1.04–0.81)
	Satisfaction With Life Scale (SWLS)		*d* = 0.67 (-0.29–1.61)
	Positive Aspects of Caregiving Questionnaire (PACQ)		*d* = -0.54 (-1.50–0.38)
	Measures of Depression (PHQ-9)		*d* = 0.00 (-0.92–0.92)
	Measures of Psychological wellbeing (Flourishing Scale)		*d* = 0.15 (-0.78–1.07)

*Effect sizes (Cohen’s *d*) were calculated on means and standard deviations pre vs. post intervention using r = .5 for correlation between measures where possible. Positive effects indicate support for hypotheses. By convention, effect sizes (Cohen’s *d*): small effect (*d* = 0.2), medium effect (*d* = 0.5) and large effect (*d* = 0.8) [51]. ^Unknown–insufficient data available to calculate 95% confidence intervals.

## Quality appraisal

The outcome of quality appraisal of quantitative studies is presented in Table 3. Inter-rater agreement was 99.1% and disagreement was resolved through further discussions. All eight studies that reported quantitative data were pilot/exploratory or feasibility studies; unsurprisingly total scores on Downs and Black were low and the range was limited (12–18 out of 28), so we did not relate quality appraisals with effect sizes. Blinding participants and assessors were difficult due to the open-label nature of intervention and study design. Attrition varied: one music study [40] reported a high attrition rate (40%), while a song writing study reported no drop-outs [43]. None of studies considered potential confounders, such as expectation of taking part in the research or prior experience of the type of arts programmes. No adverse events were reported. All studies were limited by their small number of participants. Given the pilot/feasibility nature of the studies, none of them conducted prior power calculations, except one study, which noted it was underpowered [38].

**Table 3:** Summary of quality assessment of mixed-methods studies using downs & black checklist.

Downs & Black Quality Assessment	Baker 2012 [44]	Baker 2018 [43]	Bourne 2019 [38]	Camic 2011 [42]	Camic 2014 [18]	Davidson 2014 [41]	Hanser 2011 [40]	Tamplin 2018 [39]
Q1. Hypothesis/aim/objective	1	1	1	1	1	1	1	1
Q2. Main outcomes	1	1	1	1	1	1	1	1
Q3. Characteristics of the participants	1	1	1	1	1	1	1	1
Q4. Interventions	1	1	1	1	1	1	1	1
Q5. principal confounders ^	0	0	1	0	0	0	0	0
Q6. Main findings	1	1	1	1	1	1	1	1
Q7. estimates of the random variability	0	1	1	1	1	1	0	0
Q8. adverse events	0	0	1	1	1	0	0	0
Q9. patients lost to follow-up	0	1	1	0	0	1	0	0
Q10. probability values	0	1	1	0	1	1	1	0
***Reporting (max subscore of 11)***	5	8	10	7	8	8	6	5
Q11. Study participants being representative of the entire population	0	0	0	1	0	1	0	1
Q12. Subjects who were prepared to participate representative	0	0	1	1	0	1	1	0
Q13. Staff, places, and facilities representative	0	0	0	0	1	1	1	0
***External Validity (max subscore of 3)***	0	0	1	2	1	3	2	1
Q14. Attempt made to blind participants	0	0	0	0	0	0	0	0
Q15. Attempt made to blind assessors	0	0	0	0	0	0	0	0
Q16. any the results of the study were based on “data dredging, was this made clear	1	1	1	1	1	1	1	1
Q17. Same time period for intervention and control groups	1	1	0	1	1	1	1	0
Q18. Appropriate statistical tests	1	1	1	1	1	1	1	1
Q19. Compliance with the intervention	1	1	1	0	1	1	1	1
Q20. Valid and reliable main outcome measures	1	1	1	1	1	1	1	1
***Internal Validity–Bias (max subscore of 7)***	5	5	4	4	5	5	5	4
Q21. Recruited from the same population	1	1	1	0	0	1	1	1
Q22. recruited over the same period of time	1	1	1	1	0	1	1	1
Q23. participants randomised to intervention groups	0	0	0	0	0	0	0	0
Q24. intervention assignment concealed from both patients and health care staff	0	0	0	1	0	0	0	0
Q25. adequate adjustment for confounding in the analyses	0	0	0	0	0	0	0	0
Q26. losses of patients to follow-up taken into account	0	1	1	0	0	0	0	0
***Internal Validity–Confounding (selection bias) (max subscore of 6)***	2	3	3	2	0	2	2	2
Q27. sufficient power to detect a clinically important effect (*p*< .05)	n/a	n/a	0	0	0	0	0	n/a
***Power (max subscore of 1)***	n/a	n/a	0	0	0	0	0	n/a
***Total score (max score of 28)***	12	16	18	15	14	16	15	12

Scores: 1 = Yes; 0 = No/Unable to determine. Question number 5^ was scored 2 = yes; 1 = partially; 0 = no

n/a = Not Applicable for feasibility studies, which stated that they required no prior sample size calculations.

Within the eight quantitative studies, there was a lack of homogeneity of intervention types and outcome measures which prevented meta-analyses.

### Qualitative studies

Nine of the included 17 studies used qualitative approaches [29–37], which included a total of 92 caregivers. Creative interventions included in these studies were singing [29, 30, 34], song writing [37], music [33] and art programmes [31, 32, 35, 36]. The aim of this integrative systematic review was to understand the experiences of caregivers of people with neurological conditions who had taken part in creative interventions. Whilst the focus of the review is caregivers, inevitably some of the themes from papers relate to the perceptions of caregivers related to care-recipient experiences as well. Papers included in the review highlight the inherent appeal and universality of some creative interventions reported by caregivers, particularly those that involved music and art [29]. The innate appeal of these interventions was likely to encourage maintaining participation in the activities for both the caregiver and care-recipients beyond the duration of the intervention [34]. The meta-themes below present caregivers’ experiences from a range of active participation using arts (e.g., creating artwork after viewing art or writing their own songs). (Please see Qualitative Data Audit Trail in S3 Appendix.)

### Quality appraisal

The outcome of quality appraisal of 16 studies are presented in Table 4. Inter-rater agreement was 97% and disagreement was resolved through further discussions. All 16 studies employed validated approaches for data analysis, such as thematic analysis [45] and grounded theory [50]. All studies provided details on ethical issues, participants and data collection methods. Findings were also appropriately presented alongside limitations and future recommendations. Overall, all 16 studies were rated as having good quality in relation to qualitative approaches. Thus, their data were included in the data synthesis.

**Table 4:** Summary of quality assessment of qualitative studies using CASP.

1^st^ Author Year Reference #	1. Purpose	2. Qualitative Methods	3. Research Design	4. Recruitment	5. Data Collection	6. Relationship	7. Ethics	8. Data Analysis	9. Findings	10. Contribution
Baker 2012 [44]	Y	Y	Y	Y	Y	Y	Y	Y	Y	Y
Baker 2017 [37]	Y	Y	Y	Y	Y	Y	Y	Y	Y	Y
Baker 2018 [43]	Y	Y	Y	Y	Y	Y	Y	Y	Y	Y
Burnside 2017 [36]	Y	Y	Y	Y	Y	Y	Y	Y	Y	Y
Camic 2011 [42]	Y	Y	Y	Y	Y	N	Y	Y	Y	Y
Camic 2014 [18]	Y	Y	Y	Y	Y	N	Y	Y	Y	Y
Camic 2015 [35]	Y	Y	Y	Y	Y	Y	Y	Y	Y	Y
Clark 2018 [34]	Y	Y	Y	Y	Y	Y	Y	Y	Y	Y
Dassa 2018 [33]	Y	Y	Y	Y	Y	N	Y	Y	Y	Y
Davidson 2014 [41]	Y	Y	Y	Y	Y	Y	Y	Y	Y	Y
Flatt 2015 [32]	Y	Y	Y	Y	Y	Y	Y	Y	Y	Y
Hanser 2011 [40]	Y	Y	Y	Y	Y	N	Y	Y	Y	Y
Hunt 2018 [31]	Y	Y	Y	Y	Y	Y	Y	Y	Y	Y
Osman 2016 [30]	Y	Y	Y	Y	Y	Y	Y	Y	Y	Y
Tamplin 2018 [39]	Y	Y	Y	Y	Y	Y	Y	Y	Y	Y
Unadkat 2017 [29]	Y	Y	Y	Y	Y	Y	Y	Y	Y	Y

Y = Yes; N = No

### Meta-themes

Following the meta-ethnography approach [28], six meta-themes were constructed, which synthesised the findings from included studies. (Table 5)

**Table 5:** Meta-themes derived from the included studies.

Main third order themes	Summary definition (translation) from 2^nd^ order themes	Articles including 3^rd^ order themes
***1) Cognitive and emotional impact of intervention participation***	This meta-theme includes themes pertaining to caregivers’ reports of cognitive and emotional impacts that intervention participation had for themselves and their care-recipients, such as cognitive stimulation and engagement, reactivating memories, and experiencing clarity of thoughts and increased rational thinking.	Baker 2017, 2018; Camic, 2014; Clark 2018; Dassa 2018; Davidson 2014; Flatt 2015; Hanser 2011; Osman 2016; Unadkat 2017
***2) Barriers and facilitators related to intervention access*, *structure*, *and the intervention delivery team***	This meta-theme includes themes related to caregivers’ expectations of the interventions and their perceptions of intervention features, and the delivery team that facilitated or posed barriers to accessing and engaging with the intervention. Some examples include, the impact of the intervention setting had on participants experience of the intervention, and challenges around structuring the intervention time to work both for the caregiver and care-recipient.	Burnside 2015; Camic 2011, 2014; Clark 2018; Dassa 2018; Flatt 2015; Hanser 2011; Unadkat 2017
***3) A sense of belonging and opportunities to socialise*: *Friendships and social inclusion***	This meta-theme includes themes on factors influencing the relationship between the caregiver and care-recipient dyad and relations with others, particularly in terms of the creation of a sense of belonging afforded by opportunities to participate in the creative interventions.	Baker 2012, 2017, 2018; Burnside 2015; Camic 2011, 2014, 2015; Clark 2018; Davidson 2014; Flatt 2015; Hunt 2018; Hanser 2011; Osman 2016; Unadkat 2017
***4) Skills attainment and personal development as a caregiver***	This meta-theme relates to skills gained, personal growth, and new learning experienced and reported by caregivers. Participation in creative interventions allowed participants to have new experiences and to develop confidence to express oneself.	Baker 2018; Burnside 2015; Camic 2011, 2014, 2015; Hanser 2011
***5) Making meaning*, *acceptance*, *and changes to the caregiver and care-recipient relationship***	This meta-theme focuses on themes and caregiver experiences around how they understand and make sense of their role as a caregiver, and factors influencing acceptance of the caregiver identity. The meta-theme also focuses on acceptance of changing and anticipated changes in the relationship between caregiver and the care-recipient.	Baker 2012, 2017, 2018; Burnside 2015; Camic 2011, 2014, 2015; Davidson 2014; Flatt 2015; Hunt 2018; Unadkat 2017; Osman 2016; Clark 2018; Hanser 2011
***6) Perceived positive benefits of engaging with the intervention***	This meta-theme relates to positive and uplifting experiences and benefits reported by caregivers as a result of engaging with the interventions that have an intra-individual level focus on caregivers. Experiences including improved personal wellbeing, self-esteem, positive affect, enjoyment, relaxation, feeling energized, empowered, and having confidence were captured in this meta-theme.	Baker 2012, 2017, 2018; Burnside 2015; Camic 2011, 2014, 2015; Davidson 2014; Flatt 2015; Hunt 2018; Unadkat 2017; Osman 2016; Clark 2018; Hanser 2011; Tamplin 2018

#### 1. Cognitive and emotional impact of intervention participation

This meta-theme focuses on the cognitive and emotional impact that the intervention participation had on themselves and care-recipients. The cognitive impact of intervention participation included cognitive stimulation and engagement [29, 34], through the practice of previously acquired skills (such as demonstrating musical knowledge). Music-based interventions were viewed as an opportunity for caregivers and care-recipients to demonstrate their musical knowledge, which was evidence of their cognitive abilities, and the demonstration of memory and recall over the weeks was encouraging for caregivers to see with care-recipients. Creative interventions were useful for reactivating memories and afforded opportunities for memory recall of events [18, 30, 34, 40]. Engagement in creative interventions reportedly improved care-recipients' mental capacity, according to some caregivers:

“She would be coming back (from the gallery) a bit more mentally sharp, a bit more with it on those days” (p. 165, [18]).

Creating spaces for dyads to reminisce was reported as having a positive cognitive impact on care-recipients’ memory [40]. In some cases, the meaning behind song lyrics evolved with the carer journey, with the song resonating and created an emotional response upon hearing the song [37, 43]. The process of creating a ‘time capsule’ database of music and photos from couples’ lives evoked strong positive feelings and led to improved quality time for caregivers and their care-recipients [33]. Themes related to increased lucidity, where caregivers reported clarity of thoughts and increased rational thinking, both for themselves and care-recipients were also included [41]. Some papers reported that caregivers found singing allowed them to focus and concentrate on the task of singing and develop present moment awareness, allowing them to let go of other distressing or negative thoughts [29, 41].

#### 2. Barriers and facilitators related to intervention access, structure, and the intervention delivery team

Prior to joining the interventions, caregivers had deliberations around what to expect [42], including assumptions that they had nothing to contribute to the intervention [43]. The values and perspectives of the staff and those involved in the delivery of interventions were also pertinent to participants’ experience of the interventions. One paper found that caregivers in an intervention considered themselves ordinary users of a community place that was “somewhere different” and valued as a special place [35].

The structure, ambience and environment of an intervention was key to shaping and facilitating participants’ experiences [18, 34, 40, 42]. For example, singing as part of a group was reported as an enabler for participation in interventions aimed at caregivers and care-recipients [29, 34] and an art gallery setting created an empowering space for participants [40]. One caregiver stated their views about their perceptions of their partner with dementia who also participated in the intervention:

“…she loves the looking. One of the things she mentioned a number of times is how important it is, the silence at the beginning, where they really get a chance to look. And I think that for people with… slow processing skills, not poor but slow, that element is just so important.”” (p.7 [36])

It may have been difficult to structure and time the delivery of an intervention to appeal to both caregiver and care-recipient, as illustrated by this quote:

‘‘I think it drew him out more than it drew me out. And why that is, I don’t know.” (p.8 [36])

One paper reported concerns from caregivers around the activities and logistics around the design of activities for older adults with dementia suggesting these practical aspects of creative interventions, if not developed with consideration for specific groups, could hinder participation from some potentially eligible participants [32]. In one study, participants were critical of the intervention protocol, as some caregivers found it difficult to engage the care-recipient or experienced frustration with the care-recipients' lack of focus, suggesting there were lessons to be learned for future enhancements of the intervention [40]. One key recommendation was to engage the assistance of a qualified music therapist, who is trained in adapting an intervention according to individual needs and preferences, including addressing behavioural challenge, thus affording flexibility in structure which was seen as key to caregiver satisfaction and continued participation in the intervention. Another paper reported challenges experienced by caregivers around creating a time capsule and visiting the nursing home due to communication barriers with the care-recipient. Visits were reportedly more feasible once the database had been created, which was considered helpful to caregivers [33].

#### 3. A sense of belonging and opportunities to socialise: Friendships and social inclusion

This meta-theme focuses on factors influencing the relationship between the caregiver and care-recipient dyad and relations with others, particularly in terms of the creation of a sense of belonging afforded by opportunities to participate in the creative intervention [18, 32, 34–37, 40–44]. As a result of participating in the intervention together, improved communication was reported by caregivers, not only within the dyads but also with other participants in the interventions and family members [42]. Additionally, papers reported other social benefits, including enhanced spousal relationship and strengthened reciprocity between caregiver and care-recipient [29, 35, 44].

A non-judgemental group approach was considered important to enable caregivers to voice their experiences with willing listeners [37], to share joy and sadness [29] and to feel valued by others [18]. Furthermore, in a singing intervention, the social proximity of caregivers and their care-recipients to other caregivers and care-recipients were highly valued [41].

The songs represented a shared experience, and this shared experience and collaboration was considered important [37]. Papers highlighted the socio-emotional connection afforded by participation in weekly creative interventions, which gave both caregiver and care-recipient something to look forward to attending and be part of [32, 41]:

“But this is…this is art. This is who we are. You know? And its rich and…and…people with Alzheimer’s having that. And…you know the feeling that you’re…you’re part of the whole thing too” (p.7, [36])

Papers presented themes around caregivers’ and care-recipients’ appreciation of a time and space afforded by the creative interventions to enable greater communication, and a natural development of social interactions [34, 36] both with the caregiver and other participants in the interventions. Meaningful connections made with care-recipient through the creation of art allowed caregivers to build social connections that were based on mutual interests rather than the caregiving [31]. Baker [43] reported that the song writing group filled a gap for caregivers that were not met by other support groups.

#### 4. Skills attainment and personal development as a caregiver

This meta-theme relates to skills gained, personal growth [35, 36, 43], and new learning [18, 42] experienced by caregivers. Participation in creative interventions allowed participants to have new experiences and to develop confidence to express oneself [37]. For example, as one participant stated:

“It probably unloads a lot of the… all the emotional feelings that you do hang on to that you don’t even realise are there sometimes so it helps to get them out… it was good and it was cathartic. Even though I say I don’t hold a lot you probably do have a bit of baggage and just talking about it and bringing out all those different words was, good and just very satisfying.” (p.13, [37])

In one song creation intervention, the process and product were reported to exceed caregivers’ expectations [32], and an art-viewing intervention allowed caregivers to view art in a different way [36].

One study reported an increase in household music making and listening as a result of participation in the intervention, which improved home-life for the dyad [40]. In an arts intervention, papers reported caregivers feeling more competent as a result of the skills they developed by engaging in the intervention [30]. Three papers reported the recognition some caregivers had on the importance of mindfulness and living in the moment, and that these interventions created the space for this to be experienced [29, 36, 41].

#### 5. Making meaning, acceptance, and changes to the caregiver and care-recipient relationship

This meta-theme focuses on how caregiver understand and make sense of their role as a caregiver, and the factors influencing acceptance of the caregiver identity. The meta-theme also focuses on acceptance of changing and anticipated changes in the relationship between caregiver and the care-recipient [18, 30, 31, 35, 43].

The intensity of the care-giving experience was described as a relentless battle, whereby caregivers reported grieving and feeling helpless about their loved one’s deterioration and loss [31]. Caregivers talked about the burden of responsibility, feeling out of control, losing touch with the outside world, and having to put on a brave face, even when feeling emotionally engulfed and exhausted [31]. This caused concern about the impact of caregiving on their own health [31]. For some caregivers this led to a perceived erosion of pre-caregiver identity and the creation of a new identity as caregiver, where personal interests where sacrificed, and some felt pushed to their limits of endurance [31]. Some caregivers experienced role reversal as they took on a supervisory role regarding the care-recipient, which presented challenges as well as creating stronger relationships between dyads for some [31]. Creative interventions that allow for self-expression offer opportunities to caregivers to reclaim and transform their identity [31]. Art-making was seen as a means to cope and practice resilience, where this approach allowed caregivers to make the most of their lives and to deal with an uncertain future [31]:

“It slightly reminds me of the turmoil but at the same time it says to me yes, you got through it… Because they [a series of paintings] actually were part of the process of me becoming well again.” (p. 37, [31])

Participation in creative interventions allowed for caregivers to view the care-recipient in a different light [30, 35, 37], which led to increased satisfaction with caregiving role [29, 35, 44]. Caregivers found that participating in a creative intervention provided them with insights into their own caregiving situation, which resulted in developing new perspectives on caregiving [37].

Studies reported relationships becoming normalized and allowing for positive identity construction; caregivers felt participation in the creative interventions allowed for interactions that were more equal and person-centred, as opposed to the usual caregiver–care-recipient dyad experiences that tended to occur outside the intervention [29, 36, 41]. Some papers also reported on the relationship affirming and developing qualities of participating in the intervention, as it created a space for caregivers to be with care-recipients to experience new things together in the intervention [29, 34, 36, 41].

#### 6. Perceived positive benefits of engaging with the intervention

This meta-theme relates to the positive and uplifting experiences and benefits caregivers experience as a result of engaging with the intervention that have an intra-individual level focus on caregivers. Experiences including improved personal wellbeing [29, 31, 32], self-esteem [32, 34], positive affect [30, 32, 33, 35], enjoyment [34, 40, 44], relaxation [41, 44], feeling energized [41], empowered [18] and having confidence [32] were captured in this meta-theme.

Caregivers felt it was important to have their say, and in taking the opportunity to express themselves, they felt they had become more visible [37]. Unloading emotions with others was considered cathartic [37, 43] and therapeutic [29].

In an intervention where caregivers and care-recipients met to create a database, this evoked positive feelings and hope for the caregivers [33]. In a singing intervention, one participant described their positive experiences as follows:

“Singing is a ‘chill pill’. I couldn’t believe my luck when I saw the group advertised. It’s been so lovely. It is really tough caring for Dad, but coming here and singing, well it just washes all my troubles away. I feel so calm afterwards. I reckon that I need it much more than he does.” (p.33, [41])

Several papers described the opportunity for joint respite for the caregiver and care-recipient dyad to focus on the experience of the creative intervention, without needing to focus on the condition [26, 30, 31]. The intervention activities were opportunities for playful experimentation, which was deemed the antithesis of caregiving, bringing about restorative feelings to caregivers and care-recipients [31]. The caregivers experienced an increase in positive mood when they saw care-recipients expressing happiness due to participating in the intervention [29, 34, 41, 42].

Interestingly this meta-theme did not mention the reduction of negative emotions and instead highlighted the increase in positive ones. This suggests the negative feelings were not removed by participation in creative caregiving interventions; however, caregivers and care-recipients experienced positive changes as a result of participation in these creative interventions.

## Discussion

### Summary of overall findings

The current review aimed to consolidate research findings to date, to identify gaps in current knowledge, and guide future research, practice and policy concerning the applicability of creative interventions for caregivers of people with neurological conditions. Through comprehensive and extensive searches, we found that most studies were qualitative in nature and mainly targeted dementia as the condition of focus, and there are relatively fewer creative interventions for other neurological conditions. There were a range of creative arts interventions identified, with most involving music/singing and art.

The main findings from the quantitative analysis identified that there is a lack of larger studies and randomised controlled trials. All included quantitative studies were pilot/feasibility studies employing a pre- and post-test design and had small sample sizes. Only two studies included comparisons by offering choices to participants: a song writing programme was compared with standard care [43], and group singing with art viewing [38]. Studies also varied in relation to the type of creative intervention and evaluation methods, which precluded meta-analysis. Large effect sizes were detected in wellbeing measures following singing [38, 41] and music interventions [40], however, these were not consistent across measures or studies. None of the studies examined mediator or moderator variables, and no adverse events were reported.

The qualitative synthesis highlighted that creative interventions were useful in creating a space for caregivers to make sense of, accept, and adapt to their identity as a caregiver. Joint caregiver and care recipient participation in the creative interventions also brought about positive changes to the dyadic relationship. Personal developments, such as learning new skills and having new experience through creative art making activities, were highlighted by caregivers as a positive aspect of taking part in these interventions. They also welcomed the opportunity to gain existing or new cognitive and behavioural skills, as well as having a space to unload emotions [18, 35, 36, 42, 43]. Caregivers reported both barriers and facilitators related to intervention access, structure, and the intervention delivery team, not only from their own perspective but also on behalf of their care-recipients. Group creative interventions were particularly useful in creating social ties with other caregiver and care-recipients.

### Implications

Caregivers of people with dementia were the only group targeted for the creative interventions included in this review. Given the prevalence of dementia and increased life expectancy [2], the focus on this condition within the review literature is expected and welcomed. Further exploration of the benefits of creative arts interventions for caregivers of non-dementia related neurological conditions are warranted. While the current review focused on active participation in creative arts programmes, previous studies reported that passive arts interventions (e.g., viewing art works [20], or listening to music [52]) were beneficial, so future work could compare the effectiveness of passive and active creative interventions. Moreover, the findings of the current review open the way for future research to determine whether condition-specific adaptations are needed to make creative interventions accessible and suitable for caregivers of other neurological conditions.

The review literature indicated a range of creative activities implemented in the studies. Music-based interventions for caregivers of people with dementia were the most commonly used interventions within the corpus of review studies. To a lesser extent, some interventions combined two forms of creative interventions, such as music and arts [38]. Some studies provided joint activities for the person with dementia and their caregiver, which were administered by therapists or trained facilitators [29, 34, 39, 40, 43, 44]. In most cases, studies reported outcome measures of both the people with dementia and their caregivers. Additionally, caregivers had a number of roles within the creative interventions: they encouraged the care-recipient to engage in the creative programme; they provided assessments of the care-recipient, who may have been unable to reflect on their own experiences due to advanced dementia [29, 30]; and in one study caregivers administered a home-based music programme after receiving a training session [40]. Thus, caregivers were both receiving the creative intervention and supporting their care-recipients, so that they could receive it too. In the home-based music study, 6 dyads of 14 recruited dropped out mainly due to too much burden on caregivers. This may reflect that creative arts programmes need to focus on relieving caregivers’ responsibilities, given the nature of being a caregiver is having multiple roles round the clock. Having caregivers as the programme co-facilitators may also further complicate the attempt to measure the effects of creative programmes.

There are helpful theories in understanding the benefits of participating in creative arts interventions. Flow theory by Csikszentmihalyi suggests that taking part in creative activities facilitate being in a flow state [7], which enhances mental wellbeing [11]. Additionally, frameworks linking creativity with resilience are discussed in the Introduction of this paper [13]. Moreover, Camic and colleagues endeavoured to develop a theoretical understanding of how the process of viewing and making art impacts people with dementia and their caregivers in the public art gallery context using the grounded theory approach [35]. According to their findings, the emerging theory in the context of dementia and art viewing and making has four critical factors that can influence the experience of people with dementia and their carer: valued place, intellectual stimulation, social interaction and changed perceptions. Our themes of sense of belonging, and cognitive and emotional impact concur with two of these factors. Accordingly, we support Camic’s recommendation that these four factors should be tested using robust experimental research designs [35].

However, our qualitative themes highlighted the importance of some additional factors, including enhanced positive emotions, engagement in meaningful activity, and accomplishments, along with a sense of belonging. Thus, our themes better accord with the PERMA model of positive psychology developed by Seligman which consists of four pillars linked to well-being: positive emotion, engagement, relationships, meaning and accomplishments [53]. The current review’s qualitative synthesis found that creative interventions involving singing, song writing, art viewing and making can improve wellbeing and provide benefits for caregivers in a range of ways from enhancing positive emotions to finding meaning and purpose (Table 5). This provides encouragement to researchers and practitioners to continue implementing and evaluating multiple pathways to benefit caregivers using creative interventions.

### Recommendations for future research

Creative interventions are complex in the nature and systematic approaches are needed to develop and evaluate them. Whilst the statistical significance is weak due to small sample sizes, there is preliminary quantitative evidence on singing, song writing, and art viewing combined with art making for informal caregivers of people with dementia. Building on the pilot data, future studies in these areas should further refine the intervention and determine effect sizes of outcome measures in order to adequately test their efficacy in randomised controlled trials. In relation to other creative interventions, such as dance, drama, there is a paucity of research evidence involving informal caregivers. For these interventions, more pilot studies are needed to understand what would benefit caregivers and how best the programme can be designed and delivered. Using the recent UK Medical Research Council guidance on how to develop an intervention will enable creative arts researchers to take appropriate actions and approaches that will benefit caregivers [54]. The key actions include careful planning of the intervention development process, involvement of stakeholders including caregivers and care-recipients, drawing on existing theories such as Seligman’s PERMA model [53] and Camic’s critical factors [35], and using diverse research and dissemination methods such as using the art created to understand and communicate benefits (e.g., [55]) along with assessments of acceptability, feasibility and effectiveness.

We also recommend that future studies include a very clear description of the creative programmes, practitioners delivering the programmes, and delivery modes. Reviewed studies often lacked description of specificity of the creative activities although there were notable exceptions [see 39, 40, 42]. Detailed description of the programmes alongside outcome measures will promote replication and help further develop, refine, and evaluate the intervention. In addition to documenting components of the intervention, it is important that treatment fidelity is described in order to understand process and treatment effects [56, 57].

Including research participants’ perspectives is becoming increasingly important and, in some cases, a necessary requirement [58]. Adopting a co-production approach to future interventions, where the creative arts intervention team includes caregivers and care-recipients from the development phase would inform the acceptability and usability of the intervention. For example, in one of the reviewed studies [40], participants were critical of the verbal portion of the protocol, which could have been overcome by involving potential participants during intervention development. It would be informative to understand caregivers’ preferences for where and how interventions are developed as well as their preference of delivery format, e.g. group-based, or home-based. Hanser [40] demonstrated that home-based creative programmes could be feasible; these could be particularly appealing to some caregivers who may prefer not to travel due to the nature of the neurological conditions of their care-recipient. Delivering creative programmes virtually should also be considered, particularly when travel is difficult or prohibited due to COVID-19. For example, a recent study of touchscreen-based art intervention suggested that art interventions designed for people with dementia and their caregivers can be provided using a tablet computer to promote wellbeing [20]. Thus, technology may help deliver creative interventions to caregivers, and may overcome the challenges for people who might be less mobile or have limited time due to the demands of caring. It is also worth remembering these implications are drawn from creative intervention studies that focused on experiences of informal caregivers of people with dementia and did not include other neurological conditions.

### Strengths & limitations

The current review identified gaps in the evidence of creative interventions for informal caregivers. There are encouraging preliminary data on music (singing and song writing) and art (viewing combined with making art); however, there were little data on other art forms, e.g., drama, dance. Given the included studies were pilot, or feasibility studies with a small number of participants, there is a need for well-designed experimental studies, which can provide better estimates of effects.

It was not possible to conduct meta-analysis due to limited, heterogeneous evidence; however, there were more studies that provided qualitative evidence that enabled meta-synthesis. These themes aligned with theoretical perspectives that were largely absent in the reviewed studies and which should be used in future research.

## Conclusion

This systematic review found preliminary evidence that creative arts interventions are beneficial for informal caregivers of people with dementia based on 17 included studies and mainly qualitative data. Creative interventions may appeal to caregivers, offering a range of psycho-social benefits that accord with theoretical frameworks, in particular PERMA [53]. We recommend health professionals consider creative interventions as a viable option for caregivers of other neurological conditions, alongside interventions for caregivers of people with dementia. Future research on creative interventions for caregivers of people with neurological conditions is warranted. A systematic co-production approach that involves caregivers to develop, implement, and evaluate creative interventions for informal caregivers is also recommended.

## Supporting information

S1 Checklist (DOC)

S1 Appendix Definition of a creative arts intervention. (DOCX)

S2 Appendix Search strategies. (DOCX)

S3 Appendix Audit trail of qualitative data. (PDF)

S4 Appendix Quantitative data. (DOCX)
