# Supplementary material for: An integrative systematic review of creative arts interventions for older informal caregivers of people with neurological conditions
Source: PLoS One. 2020 Dec 7;15(12):e0243461. doi: 10.1371/journal.pone.0243461 (PMC7721165; doi:10.1371/journal.pone.0243461)
Supplement: S1 Appendix — (DOCX) [file pone.0243461.s002.docx]

**S1 Appendix. Definition of a Creative Arts Intervention**

**Definition of Creative Arts Interventions**

**Creativity**: Csikszentmihalyi defines creativity as “any act, idea or product that changes an existing domain, or that transforms an existing domain into a new one”. (Csikszentmihalyi M. Creativity: Flow and the psychology of discovery and invention. New York: Harper Collins; 1996. P.28)

**Arts**: Arts Council England define the arts as visual and performing art forms, music, dance, theatre and literature (Art Council England. Great Art and Culture For Everyone: 10-year Strategic Framework 2010-2020, 2^nd^ ed. 2013. London)

**Creative Arts Intervention**: A Creative Arts Intervention is an intervention or programme which uses *arts* (visual arts, music, dance, theatre and literature) in a *creative* way (moulding and/or making something out of existing materials) for improving health and wellbeing.
