# Supplementary material for: An integrative systematic review of creative arts interventions for older informal caregivers of people with neurological conditions
Source: PLoS One. 2020 Dec 7;15(12):e0243461. doi: 10.1371/journal.pone.0243461 (PMC7721165; doi:10.1371/journal.pone.0243461)
Supplement: S2 Appendix — (DOCX) [file pone.0243461.s003.docx]

**S2 Appendix. Search Strategies**

Using the CHIP (Context, How, Issue, Population) tool, we have developed our search strategy.

**Search Terms:**

Based on the above CHIP search strategy tool, we performed searches in the major databases (e.g., Medline, PubMed, Cochrane, PsycINFO and CINAHL).

**Search Strategy Example for EMBASE:**

1. ('creative expressive therap*' OR 'music'/exp OR 'music' OR ‘sing*’ OR 'literature'/exp OR 'literature' OR 'dramatherapy' OR 'writing'/exp OR 'writing' OR 'dancing'/exp OR 'dancing' OR 'dance therapy'/exp OR 'dance therapy' OR 'art therapy'/exp OR 'art therapy' OR 'drawing'/exp OR 'drawing')
2. ('caregiver'/exp OR 'caregiver' OR 'carer'/exp OR 'carer' OR 'spouse'/exp OR 'spouse')
3. ('social psychology'/exp OR 'social psychology' OR 'psychosocial'/exp OR 'psychosocial' OR 'wellbeing'/exp OR 'wellbeing' OR 'quality of life'/exp OR 'quality of life' OR ‘stress’ OR ‘burden’)
4. ('neurologic disease'/exp OR 'neurologic disease' OR 'dementia'/exp OR 'dementia' OR 'parkinson disease'/exp OR 'parkinson disease' OR 'huntington chorea'/exp OR 'huntington chorea' OR 'multiple sclerosis'/exp OR 'multiple sclerosis' OR 'motor neuron disease'/exp OR 'motor neuron disease' OR ‘stroke’)
5. #1 AND #2 AND #3 AND #4
