# Supplementary material for: An integrative systematic review of creative arts interventions for older informal caregivers of people with neurological conditions
Source: PLoS One. 2020 Dec 7;15(12):e0243461. doi: 10.1371/journal.pone.0243461 (PMC7721165; doi:10.1371/journal.pone.0243461)
Supplement: S3 Appendix — (PDF) [file pone.0243461.s004.pdf]

S3 Appendix. Audit Trail of Qualitative Data

Reference	Baker, Grocke, & Pachana (2012)	Baker & Yeates (2017)	Baker, Stretton-Smith, Clark, Tamplin, & Lee (2018)
	Engaging in music enhanced enjoyment and relaxation	Carers assumed they had nothing to contribute	Sharing the Whole CarerJourney: From Woe to Go (Participants described the group songwriting programme as unique and helpful in that it allowed them to share their experince of caregiving with other family carer givers.)
	Enhanced quality of spousal relationship	Song creation process and product exceeded careres' expectations	We’re Singing the Same Tune:Finding Connections (Finding a commonthread was identified as a pivotal experience for the groups,helping to foster a sense of connectedness and reduce asense of loneliness.)

Strenghtened reciprocity	Importance of a shared experience	Gaining Clarity Around the CarerJourney: Discovering We've Already Founda Way Through (Caregivers found that the songwriting process enabled themto gain clarity and insight into how well they were actuallycoping, often with quite difficult circumstances, and "discover that [they'd] already found a way through.")
Increased satisfaction with caregiving role	Collaboration was important	We All Fit Under the SameUmbrella but We're Miles Apart:Developing a Group Identity (Caregivers recognized and respected each other's similarities and differences throughoutthe songwriting process and that this respect fostered a sense of group identity.)

	Unloading emotions with others is cathartic	We Can Stand Up for Ourselves, We've Got Stronger: Fostering InnerStrength And Personal Growth (stronger sense of personal resilience and empowerment)
	The song represents shared experiences	Songwriting Groups Fill a GapNot Met by Other Support Groups (Caregivers described other carersupport groups as focusing more on either information sharingor on current day-to-day challenges and issues. This differed from the songwriting group, which allowed family caregivers to share their whole carer journey and personal thoughts, feelings andexperiences around being a caregiver.)
	The song's meaning evolves	

Sensitivity: Internal

	Carers resonated and had emotional responses to hearing the song
	About the carer journey
	Insights into their own situation
	Led to new perspectives on caregiving
	Confidence to express onself increased over time
	Permission to voice with willing listeners
	Important to have your say
	Importance of non-judgemental group members

Burnside, Knecht, Hopley, & Logsdon (2015)	Camic, Williams & Meeten (2011)	Camic, Tischler & Pearman (2014)	Camic, Baker & Tischler (2015)
Facilitation: Care partner ''...she loves the looking. One of the things she mentioned a number of times is how important it is, the silence at the beginning, where they really get a chance to look. And I think that for people with ... slow processing skills, not poor but slow, that element is just so important.''	Pre-group deliberation.	Social impact. Social aspects of group; caring relationship	Ordinary users of a community place
Engagement: Care partner ''I think it drew him out more than it drew me out. And why that is, I don't know.''	Ambience and environment	Cognitive capacities. Engagement; new learning; memory	"Somewhere different"

Mindfulness: Care partner ‘‘As they say, people with dementia, you know, really live in the moment and enjoy what they can ... take what they can from the moment.’’	Structure.	Art gallery setting. Empowering (social inclusion, feeling valuable, comparison to healthcare services); engaging with artwork; intervention structucture	A special and valued place
Joint respite: Care partner ‘‘You talk about respite, but respite is always kind of one going someplace and the other going ... as partners, going different directions. And this is kind of a respite for both of us together...that allows us not to think about what we are dealing with, but focus on something that is pretty pleasant—all of that are and what we feel about art. There is no immediate need to deal with the disease you know.’’	Social inclusion		Art as a universal interest

Relationship normalizing: Care partner ‘‘It was almost like we were just equal in the class and so therefore, our interaction, I think, was ... it was nicer. It was more like we used to be, kind of.’’	The experience of singing		Competency
Relationship affirming: Care partner ‘‘Mom’s experience is her experience and I can’t make her younger. I can’t make her have less dementia ... that not my job really. It’s just to be with her and love her. (The program) gave me permission to do just that.’’	From the carer's perspective: PWD response to the group. Social inclusion; new learning; enhanced emotions.		Engagement
Relationship growth: Care partner ‘‘It was just interesting that we saw different things and that was just kind o’ cool, yeah.’’	Impact outside the group		Carer respite and support

Sensitivity: Internal

Personal growth: Care partner ‘‘It has given us (me, and also my wife) but particularly me, some chance to experience art in a different way.’’

Socialization: Care partner "My husband is more of an introvert and those casual social relations where he has to connect with people are pretty challenging for him. It was very helpful to have the ... to have the time together focused on an activity, so it didn't ... you know, it wasn't a matter of generating social interaction, but it came more naturally."

Interaction

Positive affect

Change/anticipated change

Different view of person with dementia

**Clark, Tamplin, Baker (2018)**	**Dassa (2018)**	**Davidson & Almeida (2014)**
Therapeutic Facilitation and Design. The Intervention Included Supportive Therapeutic Features That Enhanced Participants' Experiences. sub themes: structure, facilitation	visiting the (nursing home) before the intervention (creating a time capsule) was difficult and communication barriers were evident.	Lucidity: 'Looking across these sessions, I've noticed us [PWD and themselves] both being more clear, and even more rational after the sessions. That is a big difference and I'd say the impact of working over the weeks.'
Accessibility. TherapeuticSinging Group Made Singing MoreAccessible for PwD and Their FCG. Sub themes: encouraging and accepting, continuing singing, new experiences, singing is valuable, sustainability,	Creating the Database Evoked StrongFeelings and Led to Anticipation for theMutual Meetings	Energy: 'I'm astonished how these sessions have touched me. I can't really explain why, but I feel much more energetic after singing, which is good, as we often have lunch before coming along so I would normally feel like a snooze.'

Empathic Friendship.Therapeutic Group Singing FosteredNew Supportive Friendships for PwD and FCG. Sub themes: affinity, ripple effects,	The Mutual Meetings Using the DatabaseEvoked Positive Feelings and Hope	Focus: Yes, singing lets me focus. I just concentrate on what I've got to do. It is a time when I can let everything else go from my mind.
PwD/FCG Relationship.Therapeutic Group Singing SupportedRelationships Between PwD and FCG. Sub themes: togetherness, mutual benefits	Visiting After the Intervention WasPerceived as Feasible With the Help of theDatabase	Mood: 'It's marvellous to see him happy, and it lifts my spirits too. Each week it seems to get better. We both look forward to coming.'

Personal Wellbeing.Therapeutic Group Singing Led toPositive Individual Experiences for PwDand FCG. Sub themes: confidence, self idenity, mental stimulation, enjoyment, evokes memories		Relaxation: ‘Singing is a ‘chill pill’. I couldn’t believe my luck when I saw the group advertised. It’s been so lovely. It is really tough caring for Dad, but coming here and singing, well it just washes all my troubles away. I feel so calm afterwards. I reckon that I need it much more than he does.’
		Positive identity construction: ‘The thing I’ve noticed about this experience is how it shows him in a good light. I often look at him and think “that’s not the man I married”. But then since I’ve been hearing him singing and looking and behaving like a normal man and I think “that’s him, he’s still my husband in there.’”
		Socio-emotional connection: It is remarkable, the singing has been remembered from week to week and today we were really eager to get here. I’m so pleased the group is continuing. You can make friends through this sort of group.

Sensitivity: Internal

Positive identity construction: 'The thing I've noticed about this experience is how it shows<br>him in a good light. I often look at him and think "that's not<br>the man I married". But then since I've been hearing him singing and looking and behaving like a n
Proximity: 'We both enjoy the closeness. It is good to feel the others<br>huddled around, swaying with you in the music.'

Flatt, Liptak, Oakley, Gogan, Varner & Lingler (2015)	Hanser. Butterfield-Whitcomb, Kawata & Collins (2011)	Hunt, Truran & Reynolds (2018)
Cognitive stimulation: Aspects of the activity that were considered to be mentally stimulating	Increase in reminiscence	"It's twenty-four seven": the intensity of the care-giving experience. A relentless battle; the burden of responsibility; feeling engulfed and exhausted; feeling concerened about the impact of care-giving on own health
Social connections: Represents the social connections or bonding that participants experienced.	Increase in household music making or listening	"What about me?": Erosion of identity. Having to put on a brave face; care-giving as an identity marker; sacrificing personal interests; feeling pushed to the limits of endurance

Sensitivity: Internal

Self-esteem: Positive feelings or emotions expressed by participants when talking about the art engagement activity.	enjoyment of music selections	"There's nothing that can fix it": feeling helpless abou the loved one's deterioration and loss. Grieving for the loved one; feeling out of control; losing touch with the outside world.
Activity-related concerns: Aspects that seemed important for designing the activities for older adults with dementia.	enjoyment of protocol	"if we go anywhere we usually go together": impact of dementia on relationships. Taking on a supervisory role; experiencing role reversal; difficulties relating to loved on and wider family; experiencing a stronger relationship with the loved one.

Logistical issues: Planning or related issues that made the activity more feasible for participants.	other family members joined care recipient in music making or listening	"you have to play the card you're dealt": resilient changes to outlook and approach on life. Making the most of life; concerns about an uncertain future; resignation and optimistic resolve.
	criticism of verbal portion of protocol	“Like a drawing of breath”: Art-making provides a profound sense of respite from caring and confinement. Art-making…Expresses confinement and offers a means of escape Offers absorption by the creative process and sensuality of materials Restores well-being and enables recovery from care-giving Encourages playful experimentation – the antithesis of care-giving Processes the end of care-giving in rich and personal ways
	Criticism of instrument playing	“It’s part of me”: Self-expression through. Art-making Offers opportunities for choice and autonomy Reclaims and transforms identity Provides tangible evidence of coping and resilience art-making enhances identity and resilience

“Keeping that link going”: Art-making facilitates meaningful connections with a world outside of care-giving. Art-making…
Establishes a focus beyond care-giving Enables a closer relationship with the care recipient through sharing/viewing artwork together Builds social connections based on mutual interests rather than care-giving

Sensitivity: Internal

**Osman, Tischler & Schneider (2016)**	**Tamplin, Clark, Lee & Baker (2018)**	**Unadkat, Camic & Vella-Burrows (2017)**
social inclusion and support	enjoyment of the group singing programme & well accepted by the caregivers	Singing Experience. The joy of singing (Stimulating, therapeutic, enjoyment, uplifting); the accessibility of singing as an activity (Innate, Universal, Effective group facilitation, encourages participation, equality, person centred, equality as a priority, equal participation, group effects, new experiences)
a shared experience	practical elements of the intervention deliverythat participants highlighted as supportive, such as the use ofpower point display for lyrics, the size of the group, and theopportunity for social connection over afternoon tea followingthe sessions	Individual and couple benefits

positive impact on memory	Caregivers were willing to complete assessments	
positive impact on relationships	Feedback on questionnaires used for research	

lifting the spirits

acceptance of the diagnosis

Sensitivity: Internal

Sensitivity: Internal

Sensitivity: Internal

Sensitivity: Internal

Sensitivity: Internal
