# Supplementary material for: An integrative systematic review of creative arts interventions for older informal caregivers of people with neurological conditions
Source: PLoS One. 2020 Dec 7;15(12):e0243461. doi: 10.1371/journal.pone.0243461 (PMC7721165; doi:10.1371/journal.pone.0243461)
Supplement: S4 Appendix — (DOCX) [file pone.0243461.s005.docx]

**S4 Appendix. Quantitative Data**

**Quantitative Data: outcome measures and effect sizes**

| **1^st^ Author year**  [Reference number] | **Outcome measures** | **Number of**  **Participants included**  **in the analysis** | **Effect size *d****  (95% confidence intervals) | **Means (SDs)** |
| --- | --- | --- | --- | --- |
| **Baker**  **2012**  [44] | Geriatric Depression Scale Short Form (GDS)  Geriatric Anxiety Inventory (GAI)  Mutual Communal Behaviours Scale (MCBS)  Positive Aspects of Caregiving Questionnaire (PACQ)  Neuropsychiatric Inventory Questionnaire (NPI) | Music n = 5 | *d*= 0.35 (-0.92 - 1.58)  *d*= 0.45 (-0.85 - 1.65)  *d*= 0.46 (-0.82 - 1.69)  *d*= 0.25 (-1.02 - 1.47)  *d*= 0.41 (-0.84 - 1.66) | pre 5.60 (1.82) post 6.20 (1.64)  pre 6.00 (7.55) post 3.00 (2.74)  pre 37.60 (1.82) post 38.40 (1.67)  pre 41.80 (5.26) post 43.00 (4.30)  pre 28.20 (9.42) post 24.33 (9.29)  reported in table 1; page 12 |
| **Baker**  **2018**  [43] | Measures of Depression (PHQ-9) | Song writing group: n = 8  Standard care: n = 6 | Song writing group:  *d* = 0.64 (-0.50 – 1.49)  Control group:  *d* = -0.33 (-1.56 - 0.73) | pre 5.2 (5.3) post 2.6 (2.2)  pre 1.8 (1.7) post 2.5 (2.2)  *d* reported in text (and means/SDs in table 2, page 7) |
|  | Perceptions of Caregiving Experience (PACQ) | Song writing: n = 8  Standard care: n = 6 | Song writing: *d* = -0.24 (-1.30 - 0.67)  Control: *d* = -0.18 (-1.30 - 0.97) | pre 39.3 (7.7) post 36.9 (11.6)  pre 34.9 (12.1) post 32.9 (10.1)  reported in table 2, page 7 |
|  | Perceptions of Relationship with the Care Recipient (QCPR) | Song writing: n = 8  Standard care: n = 6 | Song writing: *d* = 0.14 (-0.85 - 1.11)  Control: *d* = 0.57 (-0.47 - 1.86) | pre 55.1 (7.6) post 56.1 (6.3)  pre 45.7 (7.6) post 51.0 (10.8)  reported in table 2, page 7 |
| **Bourne**  **2019**  [38] | Stress (Visual Analogue Scale) | Singing: n = 7  Art viewing: n = 6 | Singing: *d* = 0.59 (-0.55 - 1.58)   Art viewing: *d* = 0.26 (-0.88 - 1.39) | pre 62.00 (36.77) post 80.86 (26.34)  pre 70.00 (22.05) post 75.67 (20.87)  reported in table 3, page 6 |
|  | Canterbury Wellbeing Scale | Singing: n = 7  Art viewing: n = 6 | Singing: *d* = 1.16 (-0.20 - 2.00)  Art viewing: *d* = 0.60 (-0.64 - 1.66) | pre 343.14 (93.22) post 426.86 (41.69)  pre 360.17 (93.79) post 407.83 (60.60)  reported in table 3, page 6 |
| **Camic**  **2011**  [42] | Depression Anxiety Stress Scale (DASS)  – Depression                                                           – Anxiety                                                           – Stress | Singing n = 8 | *d* = 0.24 (-0.77 - 1.12)  *d* = 0.49 (-0.56 - 1.42)  *d* = -0.24 (-1.25 - 0.72) | pre 5.60 (4.50) post 4.63 (3.16)  pre 1.70 (1.64) post 1.00 (1.20)  pre 8.90 (4.70) post 10.13 (5.64)  reported in table 3, page 164 |
|  | WHO Quality of Life Questionnaire (WHO QOL_Bref)    – Physical                                                                  – Psychological                                                                  – Social                                                                  – Environment | Singing n = 9 | *d* = 0.05 (-0.89 - 0.96)  *d* = 0.13 (-0.82 - 1.03)  *d* = 0.28 (-0.70 - 1.16)  *d* = 0.20 (-0.75 - 1.10) | pre 13.96 (1.88) post 14.03 (1.07)  pre 14.36 (2.17) post 14.59 (1.22)  pre 14.67 (4.17) post 15.63 (2.36)  pre 16.76 (1.96) post 17.11 (1.56)  reported in table 3, page 164 |
| **Camic**  **2014**  [18] | Zarit Burden Interview (ZBI) | Location 1: n = 8  Location 2: n = 6 | *d* = 0.23 (unknown^)  *d* = 0.62 (unknown^) | *t*= 0.65, df = 6  *t*= 1.51, df = 4  reported in table 1, page 164 |
| **Davidson**  **2014**  [41] | Numeric Rating Scale – Positive-negative mood                                          – Energised-tired                                          – Relaxed-stressed                                          – Focused-unfocused | Singing (Group A): n = 6 | *d* = 1.29 (unknown^)  *d* = 0 (unknown^)  *d* = 0 (unknown^)  *d* = 0 (unknown^) | *t*(5) = −3.162, *p* < 0.05. No other significant differences noted. reported in text, page 7 |
| **Hanser**  **2011**  [40] | Visual Analogue Scale – Relaxation                                          – Comfort                                          – Happiness | Music n = 8 | *d* = 1.93 (0.86 – 3.28)  *d* = 1.76 (0.67 – 3.01)  *d* = 1.21 (0.27 – 2.45) | Calculated from the raw data presented in table 3, page 18-20 |
|  | Caregiving Satisfaction Scale | Music n = 8 | *d* = 0.10 (-0.32 - 0.52) | Estimated from data presented in Figure 1, page 20 |
| **Tamplin**  **2018**  [39] | Quality of Caregiver Patient Relationship (QCPR)  Satisfaction With Life Scale (SWLS)  Positive Aspects of Caregiving Questionnaire (PACQ)  Measures of Depression (PHQ-9)  Measures of Psychological wellbeing (Flourishing Scale) | n = 9 | *d* = -0.12 (-1.04 - 0.81)  *d* = 0.67 (-0.29 - 1.61)  *d* = -0.54 (-1.50 - 0.38)  *d* = 0.00 (-0.92 - 0.92)  *d* = 0.15 (-0.78 – 1.07) | pre 57.3 (9.8) post 56.2 (8.6)  pre 23.8 (6.8) post 28.3 (6.7)  pre 31.7 (9.5) post 26.4 (10.2)  pre 4.7 (5.2) post 4.7 (4.0)  pre 46.2 (6.7) post 47.2 (6.9)  reported in table 4, page 6 |

Effect sizes* (Cohen’s *d*) were calculated on means and standard deviations pre vs. post intervention using r=0.5 for correlation between measures except where noted; by convention: small effect (*d* = 0.2), medium effect (*d* = 0.5) and large effect (*d* = 0.8) [51]. ^Unknown – insufficient data available to calculate 95% confidence intervals.
